# Sprint Acceleration Mechanics: The Major Role of Hamstrings in Horizontal Force Production

**DOI:** 10.3389/fphys.2015.00404

**Published:** 2015-12-24

**Authors:** Jean-Benoît Morin, Philippe Gimenez, Pascal Edouard, Pierrick Arnal, Pedro Jiménez-Reyes, Pierre Samozino, Matt Brughelli, Jurdan Mendiguchia

**Affiliations:** ^1^Laboratory of Human Motricity, Education Sport and Health (EA6312), Faculty of Sport Sciences, University of Nice Sophia AntipolisNice, France; ^2^Laboratory Culture Sport Health Society (EA 4660), University of Franche-ComtéBesançon, France; ^3^Laboratory of Exercise Physiology (EA4338), University of LyonSaint-Etienne, France; ^4^Sports Medicine Unit, Department of Clinical and Exercise Physiology, Faculty of Medicine, University Hospital of Saint-EtienneSaint-Etienne, France; ^5^Faculty of Physical Sciences and Sport, Catholic University of San AntonioMurcia, Spain; ^6^Laboratory of Exercise Physiology (EA4338), University Savoie Mont BlancLe Bourget-du-Lac, France; ^7^School of Sport and Recreation, Sports Performance Research Institute New Zealand, Auckland University of TechnologyAuckland, New Zealand; ^8^Department of Physical Therapy, ZENTRUM Rehab and Performance CenterBarañain, Spain

**Keywords:** muscle, performance, sprint kinetics, surface electromyography, neuromuscular, isokinetics

## Abstract

Recent literature supports the importance of horizontal ground reaction force (GRF) production for sprint acceleration performance. Modeling and clinical studies have shown that the hip extensors are very likely contributors to sprint acceleration performance. We experimentally tested the role of the hip extensors in horizontal GRF production during short, maximal, treadmill sprint accelerations. Torque capabilities of the knee and hip extensors and flexors were assessed using an isokinetic dynamometer in 14 males familiar with sprint running. Then, during 6-s sprints on an instrumented motorized treadmill, horizontal and vertical GRF were synchronized with electromyographic (EMG) activity of the *vastus lateralis, rectus femoris, biceps femoris*, and *gluteus maximus* averaged over the first half of support, entire support, entire swing and end-of-swing phases. No significant correlations were found between isokinetic or EMG variables and horizontal GRF. Multiple linear regression analysis showed a significant relationship (*P* = 0.024) between horizontal GRF and the combination of *biceps femoris* EMG activity during the end of the swing and the knee flexors eccentric peak torque. In conclusion, subjects who produced the greatest amount of horizontal force were both able to highly activate their hamstring muscles just before ground contact and present high eccentric hamstring peak torque capability.

## Introduction

Sprint running, and more specifically sprint acceleration, is a key component of performance in many sports such as athletics, soccer and rugby. In the two latter, although the overall time spent by players at maximal acceleration or speed is lower than that at lower intensities (Stølen et al., 2005; Osgnach et al., 2010; Gabbett et al., 2012; Kempton et al., 2015), maximal acceleration over short distance is crucial to performing defensive and offensive key actions (Stølen et al., 2005; Faude et al., 2012).

Recently, researchers have clearly shown that the horizontal component of the resultant (i.e., total) ground reaction force (GRF) was the key mechanical feature of sprint acceleration performance, regardless of skill level (Hunter et al., 2005; Kugler and Janshen, 2010; Morin et al., 2011, 2012; Kawamori et al., 2013; Otsuka et al., 2014; Rabita et al., 2015). These authors consistently reported that the ability to orient the resultant GRF vector forward thus producing high amounts of net horizontal GRF (*F*_H_) throughout the sprint was the strongest predictor of acceleration and sprint performance in subjects ranging from recreational to world-class sprinters. Sprint acceleration performance was, however, not related to the magnitudes of vertical (*F*_V_) or resultant GRF (*F*_RES_) as quantified over the entire acceleration phase on an instrumented treadmill or directly on a track in various sportsmen including team sport athletes and elite sprinters (Morin et al., 2011, 2012; Rabita et al., 2015). Last and essentially, the difference in *F*_H_ production between non-specialists, intermediate and top-level sprinters was greater at higher running speeds (Morin et al., 2012; Morin, 2013). These results experimentally and consistently showed the mechanically logical importance of *F*_H_ production for forward motion and in turn sprint acceleration performance (Furusawa et al., 1927; Best and Partridge, 1928; Jacobs and Van Ingen Schenau, 1992).

When trying to explain the muscular origin of this efficient horizontally-oriented GRF production, previous researchers have focused on the hip extensor muscles, in particular the hamstrings, for the following three main reasons.

First, several studies based on experimental measurements (including surface electromyography, GRF, or motion analysis), modeling simulations, or a combination of these two approaches showed the important role of the hip extensors (gluteal and hamstring muscles) in running performance (e.g., Wiemann and Tidow, 1995; Bartlett et al., 2014; Schache et al., 2014). Indeed, using various levels of experimental/modeling data, and various subjects including high-level sprinters, Mann and Sprague (1980), Belli et al. (2002), Kyrölainen et al. (2005), Simonsen et al. (1985), Bezodis et al. (2008), Dorn et al. (2012), and Schache et al. (2015) consistently showed that the hip extensor/knee flexor muscle actions played a predominant role as running speed increased and reached high (>7 ms^−1^) to maximal sprint speeds. In most of these studies, this predominance was shown to occur during both swing and contact phases but was not explicitly related to concomitant direct measurements of *F*_H_.

Second, in order to produce high amounts of horizontal ground reaction force and impulse (due to reduced braking component and/or increased propulsive component Morin et al., 2015), especially at high running speeds (when the overall orientation of the body is vertical), intense backward movements of the lower limb are necessary during both stance and late swing phases with the hamstring muscles producing very high forces during both phases (Morin, 2013; Sun et al., 2015). Some authors suggested that the rate of force production during the early stance phase was a limiting factor for maximal sprinting speed (Weyand et al., 2010; Clark and Weyand, 2014), while others suggested that the ability of the knee flexors to reduce the kinetic energy of the lower limb while lengthening during the late swing phase and thus increasing stride frequency was paramount (Caldwell and Chapman, 1989; Dorn et al., 2012). Because of the overall very fast motion of the lower limb (step rates of 4 Hz or more), the transition between swing and stance is very short (typical total swing and stance times of 300 and 100 ms, respectively). In this context, recent studies have investigated the interaction (not isolation) between these two phases (Clark and Weyand, 2014) in order to maximize running speed. It has been suggested that the amount of knee elevation sprinters achieve late in the swing phase, i.e., when hamstrings are actively lengthened (eccentric force >6–8 times BW as calculated by Sun et al. (2015) and Schache et al. (2010), appears to contribute to the subsequent early stance GRF application through a reduced deceleration time during impact. Therefore, because great limb velocities prior to foot ground impact occur during sprinting, this swing-stance transition moment is of crucial importance for hamstrings, which counteract both external hip flexion and knee extension moments and support forces as high as eight times BW (Sun et al., 2015).

Finally, and interestingly, the sole fact that hamstring injuries are the most frequent lower limb muscle injury occurring during sprinting tasks presupposes the importance of this muscle group when the goal is to develop high speed and/or accelerations (e.g., Ekstrand et al., 2011; Feddermann-Demont et al., 2014). Although the exact moment of occurrence is debated (i.e., end of swing or stance phase: Heiderscheit et al., 2005; Chumanov et al., 2007, 2011, 2012; Yu et al., 2008; Schache et al., 2011; Orchard, 2012; Higashihara et al., 2014; Ono et al., 2015), most muscle strains share the sprint action as the main injury mechanism (Arnason et al., 2004; Woods et al., 2004; Ueblacker et al., 2015). Moreover, researchers aiming to quantify sprint running mechanics in athletes recovering or having recovered from a recent hamstring injury showed that one of the mechanical features of their running pattern differing from uninjured counterparts is altered *F*_H_ production (Brughelli et al., 2010; Mendiguchia et al., 2014). The main interpretation given by these authors was that hamstring muscle weakness induces, as previously reported, a weaker hip extension/knee flexion function (Sugiura et al., 2008; Opar et al., 2013; Sanfilippo et al., 2013), and in turn a lower *F*_H_ production. Mendiguchia et al. (2014) have clearly supported this line of thinking in high-level soccer players, with *F*_H_ derived from real-practice field sprint accelerations and radar measurements. In this study, the field sprint acceleration running mechanics, and especially the capability of high *F*_H_ production, were substantially impaired in players returning from a hamstring injury.

Since previous studies suggested that (i) hip extensor and knee flexor muscles actions play an important role in high running speed mechanics and (ii) an impaired hamstring function was associated with lower levels of *F*_H_ production and sprint acceleration performance, we sought to directly and experimentally investigate the role of hip extensors and knee flexors in *F*_H_ production during sprint running accelerations. In light of the aforementioned conclusions, our main hypothesis was that the highest amounts of *F*_H_ during a maximal sprint acceleration would be observed in subjects with the highest level of hip extension/knee flexion force and electrical activity.

All the previously mentioned studies had one or more of the following limitations: the use of forward simulation modeling; non-synchronization or lack of EMG, motion analysis or GRF measurements; data acquisition over only one to a few steps; only vertical and no horizontal GRF measurement; steady, not accelerated running speeds. The latter point affects all the aforementioned studies and is paramount for an accurate understanding of the role of hip extensors during accelerated sprint running (see discussion in Schache et al., 2014). Thus, in order to test our hypothesis and avoid most of the previous limitations we designed a protocol in which EMG, GRF and sagittal plane motion analysis were synchronized over entire accelerations on an instrumented treadmill. This allowed us to experimentally monitor the muscular activity, *F*_H_ production and lower limbs kinematics for all steps of maximal sprint accelerations. In addition, we measured the torque production capability of the main muscle groups involved in hip and knee flexion and extension using an isokinetic dynamometer. Therefore, the aim of this study was to seek the main muscular correlates of horizontal GRF production during sprint acceleration.

## Materials and methods

### Population

Fourteen male subjects (mean ± SD; body mass: 79.9 ± 7.9 kg; height 1.79 ± 0.07 m; age 24.2 ± 4.6 years) trained for sprint running volunteered to participate in this study. All subjects were free of musculoskeletal pain or injuries, as confirmed by medical and physical examinations. Seven subjects were physical education students and physically active, and had practiced physical activities including sprints (e.g., soccer, basketball) in the 6 months preceding the study. Three subjects were regional to national-level athletes (specialized in sprint and decathlon), and four subjects were under-23 high-level rugby union players. Written informed consent was obtained from the subjects, and the study was approved by the institutional ethics review board of the Faculty of Sport Sciences, and conducted according to the Declaration of Helsinki II.

### Experimental protocol

During the first session, subjects reported to the laboratory for medical examination performed by a medical doctor (PE) and completed a familiarization session with treadmill sprinting and isokinetic testing procedures. The second session (within a 2-week period), started with a standardized warm-up comprising 5 min of 10 km.h^−1^ running, followed by 5 min of sprint-specific muscular warm-up exercises, and three progressive 6-s sprints separated by 2 min of passive rest. Subjects then performed the isokinetic testing. After this testing, EMG electrodes were placed, and subjects performed maximal voluntary isometric contractions (MVIC) of knee and hip extensors and flexors. Then, motion analysis markers were placed, and subjects performed one more warm-up acceleration before the 6-s maximal intensity sprint. All subjects wore their usual running shoes (no athletics spikes used).

### Isokinetic muscle torques testing

Muscular dynamic strength was evaluated by use of an isokinetic dynamometer (Con-Trex® MJ; CMV AG, Dübendorf, Switzerland). For knee flexors (hamstring) and extensors (quadriceps) testing, each subject was seated on the dynamometer (with 105° of coxofemoral flexion), with auto adhesive straps placed horizontally across the chest and pelvis in order to stabilize the trunk to the seat, uniformly as described in the Con-Trex® owner's manual and in Maffiuletti et al. (2007). The axis of rotation of the knee joint was aligned with the rotational axis of the dynamometer, and the cuff of the dynamometer's lever arm was secured around the ankle, proximal to the malleoli. Subjects were also instructed to grip the seat during tests. The range of knee motion was fixed at 90° (from full extension to 90° of knee flexion). Appropriate support was provided to stabilize the contralateral lower limb.

For hip flexors and extensors (gluteus) testing, each subject laid in the supine position with the hip in the sagittal plane and the knee flexed at 90° following the method of Julia et al. (2010). The contralateral leg rested on (but was not attached to) a support under the foot, with 0° of hip extension and the knee flexed at 90° (Julia et al., 2010). The dynamometer's axis was aligned with the trochanter major (corresponding to the axis of hip flexion/extension). The subject's body was held by a strap around the pelvis (over the anterior superior iliac spines) and one chest belt. The evaluation was performed with a joint amplitude of 90° (from 10° of hip extension to 80° of flexion).

The subject's leg-segment and the testing apparatus were statically weighed to provide gravity compensation data, and corrections were incorporated (Maffiuletti et al., 2007; Julia et al., 2010). Subjects were verbally supported without visual feedback. The same examiner (PE) conducted the tests for all subjects. Only the right lower limb was tested, since it was the only side with EMG and video analyses. Hamstrings and quadriceps were assessed before hip flexors and glutei, using the same procedure of contraction mode and angular velocity described below. As a specific warm-up with isokinetic movements, each subject performed 2 series of 6 graded submaximal concentric repetitions at an intermediate angular velocity of 120°s.^−1^, followed by 3 submaximal repetitions at 120°s.^−1^ in the concentric and eccentric mode in a randomized order. Data of maximal isokinetic torque were obtained during 3 maximal repetitions at 120°s.^−1^, in concentric and eccentric mode in a randomized order. A 60-s rest separated each series of movements. This angular velocity was selected after preliminary testing since it was the highest velocity for which subjects could produce maximal force in safe and painless conditions.

Hamstring, quadriceps, hip flexors and glutei dynamic torques were evaluated using measurements of peak torque normalized to body weight (PT_BW_ in Nm.kg^−1^), in order to better account for differences in subjects morphological characteristics. The conventional ratio of hamstring-to-quadriceps concentric peak torque (H_con_/Q_con_), and the functional ratio of eccentric hamstring to concentric quadriceps peak torque (H_*ecc*_/Q_con_) were then calculated.

Reliability of each parameter was calculated using data from the first familiarization session and the second testing session following the statistical methods previously described (Hopkins, 2000; Maffiuletti et al., 2007). For hamstring and quadriceps muscles, reliability of peak torques was high (intraclass correlation coefficient (ICC): 0.86–0.95; standard error of measurement (SEM): 3.8–8.5%; and coefficient of variation (CV): 3.0–5.7%), and reliability of ratios was moderate (ICC: 0.69–0.85; SEM: 6.2–7.5%; and CV: 5.5–5.7%). For hip flexors and gluteus muscles, reliability of peak torques was moderate (ICC: 0.60–0.78; SEM: 9.6–19.4%; and CV: 8.0–17.3).

### Sprint kinetics

Sprint kinetics were measured using a motorized instrumented treadmill (ADAL3D-WR, Medical Development—HEF Tecmachine, Andrézieux-Bouthéon, France), for more details, see Morin et al. (2010). It is mounted on a highly rigid metal frame fixed to the ground through four piezoelectric force transducers (KI 9077b, Kistler, Winterthur, Switzerland), and installed on a specially engineered concrete slab to ensure maximal rigidity of the supporting ground. The constant motor torque was set to 160% of the default torque, i.e., the motor torque necessary to overcome the friction on the belt due to subject's BW. The default torque was measured by requiring the subject to stand still and by increasing the driving torque value until observing a movement of the belt greater than 2 cm over 5 s. This default torque setting as a function of belt friction is in line with previous motorized-treadmill studies (Falk et al., 1996; Morin et al., 2011, 2012). Motor torque of 160% of the default value was selected after several preliminary measurements (data not shown) comparing various torques, because (i) it allowed subjects to sprint in a comfortable manner and produce maximal effort without risking loss of balance, and (ii) higher torques caused loss of balance in some subjects, and prevented them, even after familiarization, to sprint with the same technique as on the track.

Subjects were tethered by means of a leather weightlifting belt and thin stiff rope (0.6 cm in diameter) rigidly anchored to the wall behind the subjects by a 0.4 m vertical metal rail. When correctly attached, subjects were required to lean forward in a typical crouched sprint-start position (standardized for all subjects and close to that in the field) with their preferred foot forward. After a 3-s countdown, the treadmill was released, and the belt began to accelerate as subjects applied a positive horizontal force. Mechanical data were sampled at 1000 Hz throughout the sprint, allowing determination of the beginning of the sprint, defined as the moment the belt speed exceeded 0.2 m.s^−1^. After appropriate filtering (Butterworth-type 30 Hz low-pass filter), instantaneous values of GRF and belt speed were averaged for each contact period (vertical force above 30 N), which corresponds to the biomechanical/muscular specific event of one leg push. Instantaneous data of vertical, horizontal, and total GRF were averaged for each support phase (*F*_V_, *F*_H_, and *F*_RES_, respectively), expressed in N and BW and used with the corresponding average belt speed (*S* in m.s^−1^) to compute net horizontal power (*P* = *F*_H_.*S*, expressed in W.kg^−1^).

### Muscular activity

**Figure 1:**
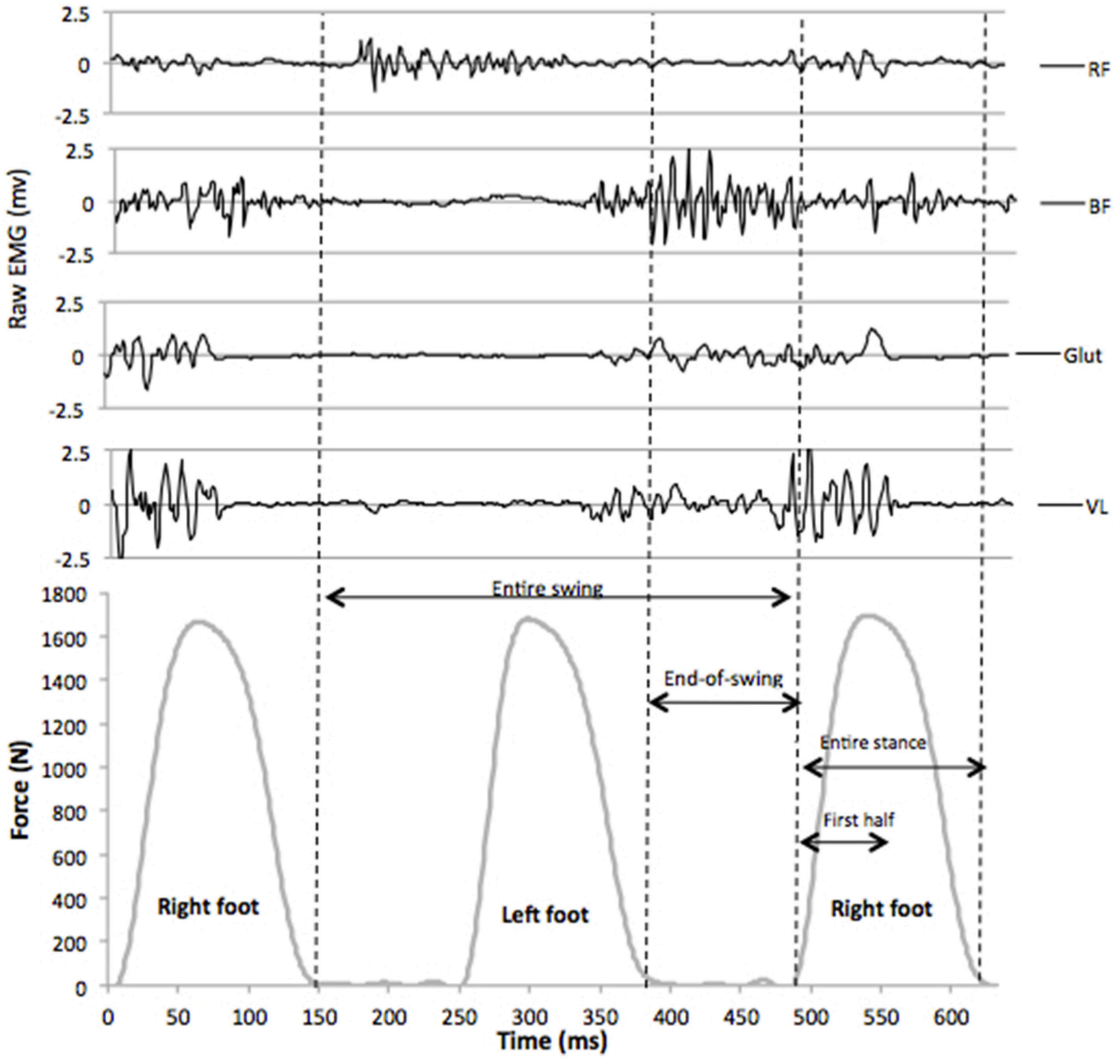
**Raw EMG signals for ***vastus lateralis*** (VL), ***gluteus maximus*** (Glut), ***biceps femoris*** (BF), and ***rectus femoris*** (RF) muscles, synchronized with vertical GRF during a typical sprint stride (7th stride of a maximal 6-s acceleration)**. The following phases for EMG analysis were determined from vertical GRF data (30 N threshold): entire swing, end-of-swing, entire stance, and first half of the stance.

EMG activity of the right *vastus lateralis* (VL), *rectus femoris* (RF), *biceps femoris* (BF), and *gluteus maximus* (Glut) muscles were recorded using bipolar silver chloride surface electrodes of 30 mm diameter (Meditrace 100, Tyco healthcare, Mansfield, Canada). The recording electrodes were taped lengthwise on the skin with respect to the underlying muscle fiber arrangement and located according to recommendations by SENIAM (Hermens et al., 2000) with an inter-electrode distance of 30 mm. The reference electrode was attached to the skin facing the patella. Low impedance (*Z* < 5 kΩ) at the skin-electrode surface was obtained by abrading the skin with thin sand paper and cleaning with alcohol. EMG data were recorded with PowerLab system (16/30—ML880/P, ADInstruments, Bella Vista, Australia) with a sampling frequency of 2000 Hz. The EMG signal was amplified with an octal bio-amplifier (Octal Bioamp, ML138, ADInstruments) with a bandwidth frequency ranging from 5 to 1000 Hz (input impedance = 200 MΩ, common mode rejection ratio = 85 dB), transmitted to the PC and analyzed with LabChart 7.3 software (ADInstruments). Vertical GRF and EMG signals for the right leg were time synchronized on LabChart 7.3, EMG activity of each muscle was quantified using the root mean square (RMS) with a 20-ms moving window, and recorded during the following phases of the running cycle for the right leg: (i) first half of the stance phase, (ii) entire stance phase as detected by a 30-N threshold, (iii) entire swing phase (from foot takeoff to the subsequent landing of the same foot), and (iv) end-of-swing phase, defined as the aerial phase (no foot-ground contact) preceding the stance phase (Figure 1). As for previous sprint studies (Jönhagen et al., 1996; Kyrölainen et al., 2005; Higashihara et al., 2010; Dorel et al., 2012; Ono et al., 2015), and according to Burden (2010), RMS data for all phases were normalized to MVIC data obtained with the following procedure.

Two 3-s duration MVICs were performed in the sagittal plane to assess hip extension (glutei muscles) and knee flexion (hamstrings) and extension (quadriceps) for the right hip and knee with a constant angle. Hip extension was tested with subjects lying on a table in a prone position at 30° hip flexion and the knee fully extended. Knee extension and flexion were tested with subjects seated in the frame of a Cybex II seat (Ronkonkoma, NY), fastened to the frame at the pelvis and with knee and hip angles set at 90°. During these three sets of 2 MVICs, two experimenters applied a firm manual resistance at the subjects' ankles to ensure a safe maximal isometric exertion.

### Sagittal plane foot motion

**Figure 2:**
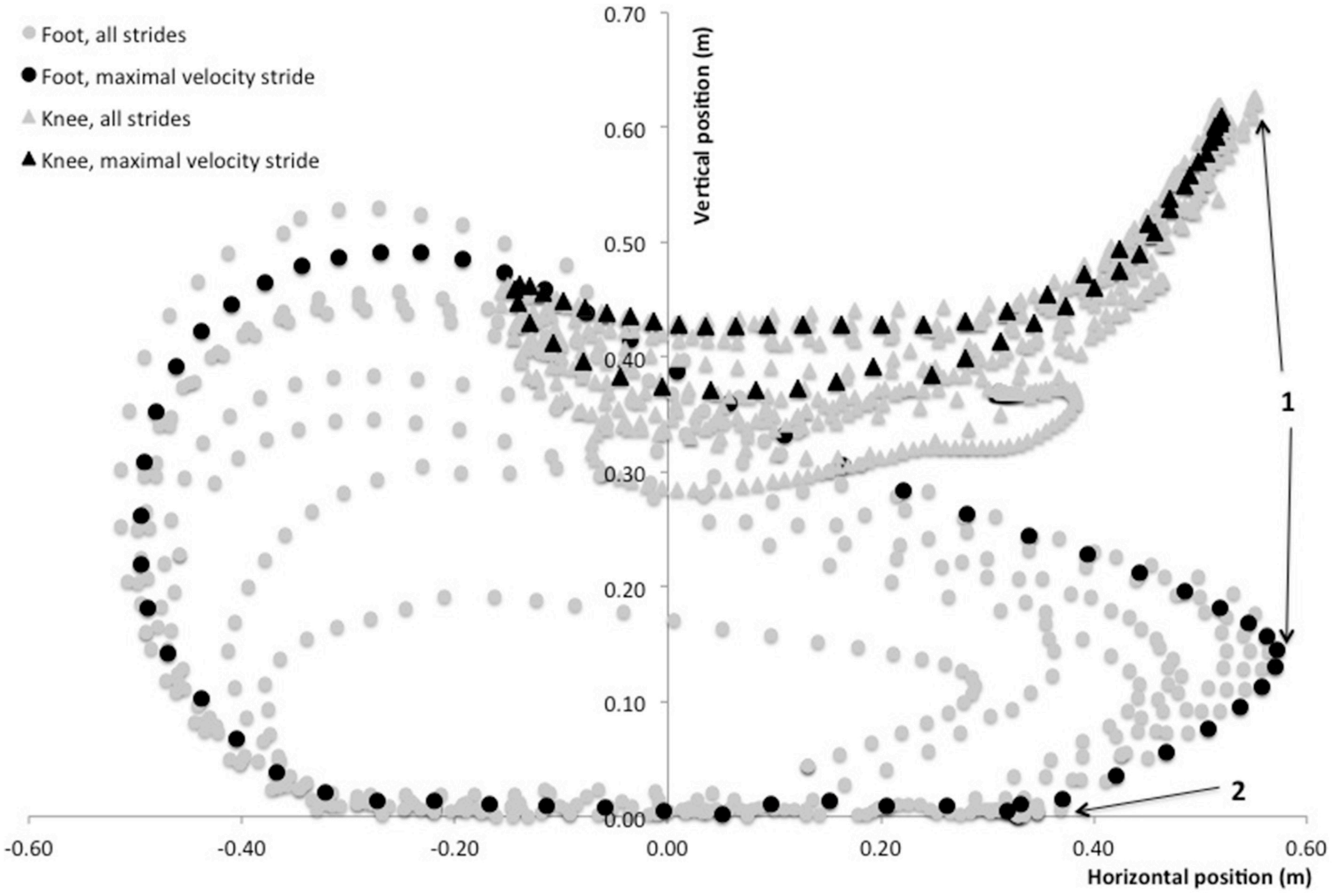
**Typical foot (circles) and knee (triangles) path diagram during an entire sprint treadmill acceleration of a rugby player**. The stride at maximal running velocity (9th stride in this trial) is shown with black circles and triangles. Positions are displayed with reference to the treadmill belt frame (vertical axis) and the subject's resting standing position was used for calibration (femoral marker in the horizontal axis). Position 1 indicates the maximal forward position of the foot and knee as retained for analyses. Position 2 indicates the initial foot-ground contact. The path of the foot marker from position 1 to position 2 is considered as the “pawing” backward motion of the foot at the end of the swing phase.

The motion of the right foot was recorded in the sagittal plane of motion with a camera (sampling rate of 120 frames per second, Basler scA640-120gc, Basler AG, Germany) mounted on a tripod placed 1.5 m away from the treadmill in a lateral view. One retro-reflective marker was placed onto the great trochanter, one onto the lateral femoral epicondyle and one marker was placed onto the external face of the shoe at the fifth metatarsal head. This point was chosen since sprint running is essentially a digitigrade action and thus subjects stroke the treadmill belt with their metatarsals first. Two other markers were placed 1.5 m apart on the right frame of the treadmill, as close to the rolling belt as possible (i.e., less than 0.5 cm). The line between these markers indicated the ground level. The video system was calibrated using a custom four-marker cross of known dimensions. After marker placement, a 3-s calibration of the video was performed during which subjects stood still with their feet parallel and arms along the body. This static position was used to define the reference ground level (vertical distance between the line delimited by the treadmill frame markers and the foot marker). Marker trajectories in the sagittal plane (vertical and horizontal directions) were tracked and analyzed with Simi Motion 2D software (Simi Reality Motion Systems GmbH, Unterschleissheim, Germany). The 2D coordinates of the foot and femoral markers allowed us to calculate foot and knee position over time during all strides of the acceleration. For clarity reasons, and given the focus of the present study on the factors related to horizontal GRF production and the importance of the preceding swing phase, the kinematic variables of interest (Figure 2) were: maximal forward and vertical positions of the foot and knee during the swing relative to the great trochanter; horizontal velocity of the foot at the end of the swing (i.e., at the last frame before foot-ground initial contact); horizontal distance covered by the foot during its backward motion (if any) between its maximal forward position and its position at initial foot-ground initial contact.

### Data analysis and statistics

Descriptive statistics are presented as mean values ± SD. Normal distribution of the data was checked by the Shapiro-Wilk normality test. In order to account for the entire sprint mechanical output, our hypothesis was tested using Pearson's correlation computed between experimental independent variables (muscular torque, muscular activity, and motion analysis), and the dependent variables of sprint kinetics (mainly horizontal GRF). Furthermore, we used enter linear multiple regressions models to test the relationship between muscular torques and the corresponding muscular activity during sprinting (as independent variables) and sprint kinetics as dependent variables. Finally, in order to better describe acceleration mechanics, some of the analyses were distinguished between the initial acceleration phase (10 first steps) and the end of acceleration phase (step 11 to the step at maximal velocity). Note that some subjects completed their acceleration within 20 steps (i.e., 10 strides for the right leg that was equipped with EMG electrodes and motion analysis markers), and all subjects had reached a velocity higher than 95% of maximal velocity at the 20th step). The significance level was set at *P* < 0.05, and statistics were performed with Sigmaplot 12.5 software (Systat Software, Inc., San Jose, CA).

## Results

The average maximal horizontal mechanical power output was 22.4 ± 2.7 W.kg^−1^, and maximal running velocity was 6.63 ± 0.61 m.s^−1^ at the end of the acceleration. This maximal velocity was reached in 20 to 28 steps (i.e., 10–14 strides for the right leg).

The main results for isokinetic muscle torques testing are shown in Table 1.

**Table 1:** **Data of hip and knee extension and flexion peak torques measured during isokinetic testing in concentric and eccentric modes**.

	**Mean (*SD*)**	**Range (Min-Max)**
**KNEE EXTENSION**		
Concentric	2.68 (0.22)	2.36–3.24
Eccentric	3.92 (0.58)	3.08–4.79
**KNEE FLEXION**		
Concentric	1.73 (0.34)	1.11–2.41
Eccentric	2.29 (0.47)	1.57–3.55
**HIP EXTENSION**		
Concentric	2.55 (0.42)	1.85–3.28
Eccentric	3.36 (1.08)	1.83–5.62
**HIP FLEXION**		
Concentric	2.28 (0.33)	1.82–2.87
Eccentric	2.97 (0.79)	2.02–4.26
**KNEE RATIOS (%)**		
Conventional	64.7 (11.8)	42.6–85.0
Functional	85.7 (16.5)	60.5–124

*All peak torque data are normalized to body mass and expressed in N.m.kg^−1^. Conventional ratio is computed as knee flexion divided by knee extension concentric peak torque. Functional ratio is computed as knee flexion torque in eccentric mode divided by knee extension torque in concentric mode*.

Sprint acceleration mechanics are shown in Table 2. A distinction was made between values averaged over the entire acceleration (i.e., from step 1 to the step at maximal velocity for each subject), and the initial acceleration and final acceleration phases (steps 1–10 and 11 to last, respectively).

**Table 2:** **Main sprint running mechanics for the entire sprint acceleration, the first (10) steps, and the last steps of the sprint, i.e., from the 11th step to the step at maximal velocity**.

	**All steps of the acceleration**	**Initial acceleration (first 10 steps)**	**End of acceleration (steps 11 to last)**
**STEP TEMPORAL VARIABLES**			
Contact time (s)	0.149 (0.014)	0.160 (0.016)	0.138 (0.013)
Aerial time (s)	0.093 (0.011)	0.084 (0.011)	0.102 (0.012)
Swing time (s)	0.333 (0.027)	0.326 (0.029)	0.344 (0.030)
Step frequency (Hz)	4.14 (0.37)	4.13 (0.34)	4.18 (0.34)
**RUNNING KINETICS**			
Horizontal force (BW)	0.350 (0.034)	0.425 (0.043)	0.278 (0.032)
Vertical force (BW)	1.62 (0.13)	1.51 (0.10)	1.73 (0.13)
Resultant force (BW)	1.66 (0.13)	1.57 (0.10)	1.75 (0.13)

*Data are presented as Mean(SD) for the group*.

**Figure 3:**
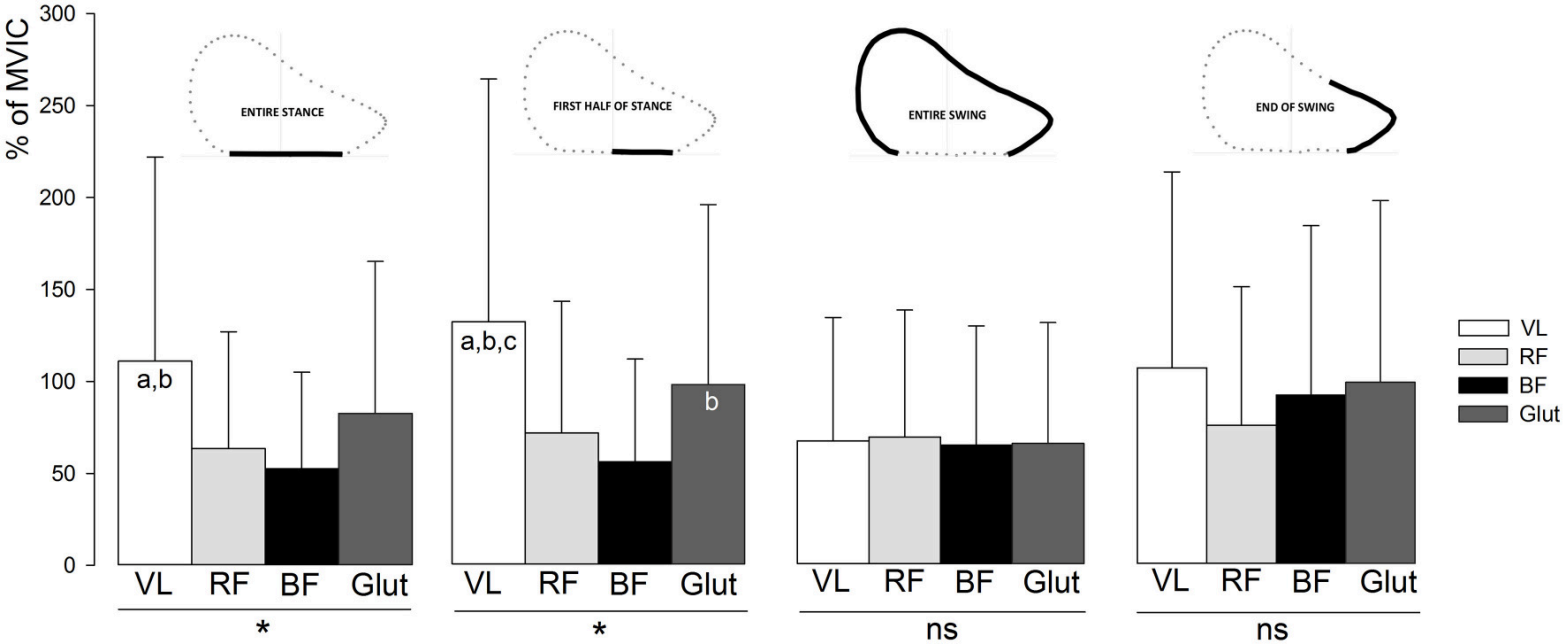
**Average EMG activity of the VL, RF, BF, and Glut muscles over the entire sprint acceleration (all steps), for the different phases of the step cycle**. Error bars indicate standard deviation. VL, *vastus lateralis;* RF, *rectus femoris*; BF, *biceps femoris*; Glut, *gluteus maximus;* MVIC, maximal voluntary isometric contraction. The muscle effect on EMG activity was tested by One-way analyses of variance (^*^, ANOVA significant main effect with *P* < 0.05; ns, not significant) and Newman Keuls *post-hoc* tests (a, significantly different from RF; b, significantly different from BF; c, significantly different from Glut).

**Figure 4:**
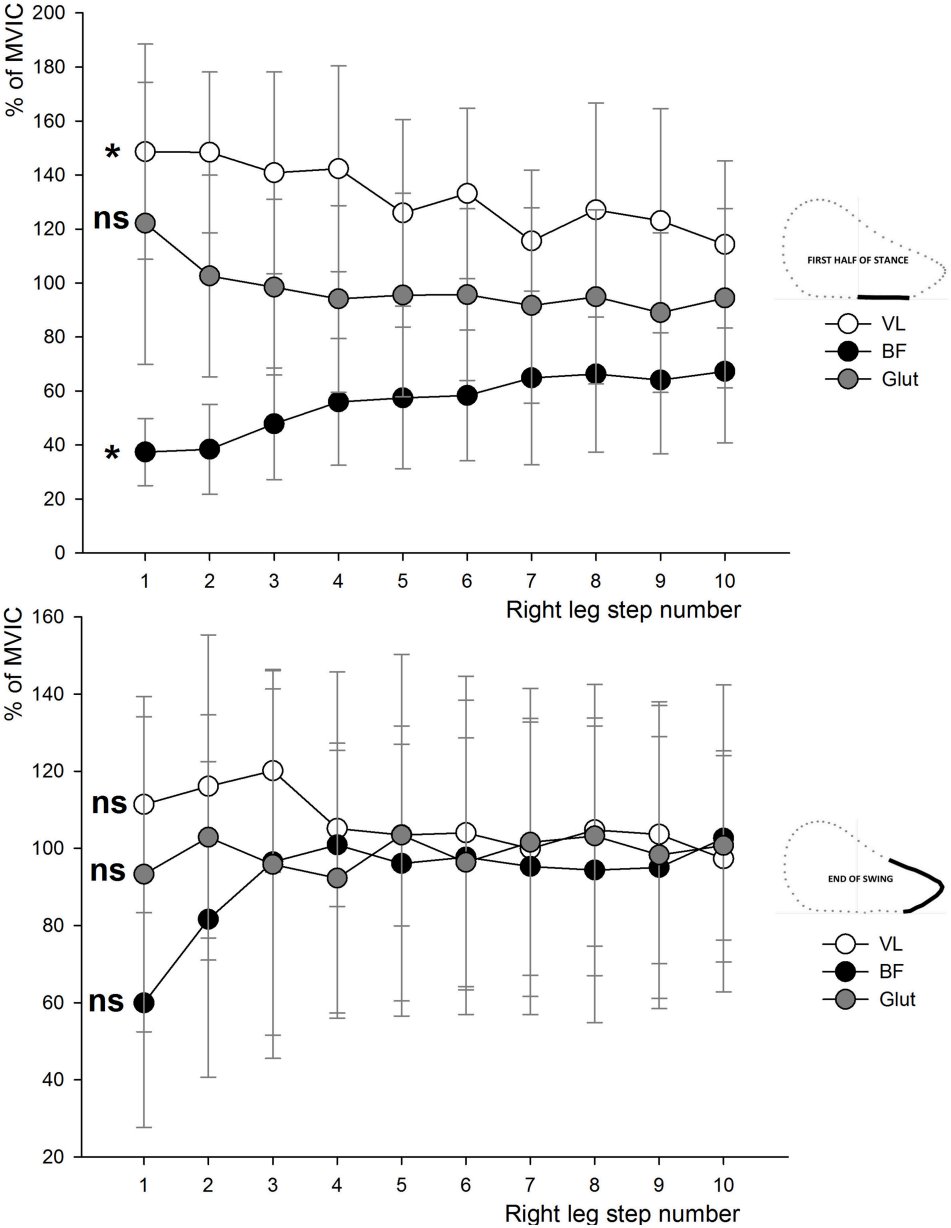
**Average EMG activity (±SD) of the VL, BF, and Glut muscles during the first half of the stance and the end-of-swing phase for all the right leg steps of the sprint acceleration**. VL, *vastus lateralis*; RF, *rectus femoris*; BF, *biceps femoris*; Glut, *gluteus maximus*; MVIC, maximal voluntary isometric contraction. The effect of “step number” over the entire acceleration were tested by One-way analyses of variance: ^*^, ANOVA significant main effect (*P* < 0.05); ns, not significant.

EMG activity of the VL, BF, RF, and Glut muscles are shown in Figures 3, 4. These descriptive figures show that the relative activities of the hip extensors and knee extensors differ between the phases of the sprint step cycle (Figure 3) and between the different steps of the sprint acceleration (Figure 4). In the latter, EMG activity of the BF during late swing and first half of contact phases increases over the acceleration, whereas that of the Glut and VL muscles tend to decrease or remain constant.

The motion analysis of the knee and foot in the sagittal plane showed that the maximal forward positions of the foot and knee relative to the great trochanter during the swing were 0.395 ± 0.075 and 0.337 ± 0.032 m, respectively. These maximal forward positions corresponded to vertical positions (taking the ground level as reference) of 0.167 ± 0.036 and 0.610 ± 0.037 m, respectively. The backward horizontal velocity of the foot at the end of the swing (i.e., just before foot-ground initial contact) was 5.27 ± 0.77 ms^−1^ (grand average value for all steps in all subjects, range: 3.53–6.72). The horizontal distance covered by the foot during the backward motion between its maximal forward position and its position at initial foot-ground initial contact (both relative to great trochanter position) was 0.244 ± 0.075 m (grand average value for all steps in all subjects, range: 0.098–0.413).

None of the isokinetic variables measured were significantly correlated with *F*_H_. Furthermore, EMG activity was not significantly correlated with *F*_H_, whatever the muscle group and the part of the running cycle considered. Only a non-significant tendency (*P* = 0.074) was found between EMG activity of the BF over the end-of-the-swing phase and *F*_H_, both averaged over the entire acceleration (i.e., all steps from the first to the step at maximal velocity).

When considering data from all steps of the acceleration, the multiple regression analysis showed a significant (*P* = 0.024) relationship between *F*_H_ and the combination of both EMG activity of the BF during the end-of-swing phase and knee flexion peak torque in eccentric mode (Table 3). A tendency toward significant relationship was found when considering knee flexion peak torque in concentric mode (*P* = 0.045, Table 3). No other significant multiple regression was found when considering data averaged over all steps of the acceleration.

**Table 3:** **Multiple linear regression analysis for horizontal ground reaction force (in BW) averaged over the entire acceleration (dependent variable in both models), ***n*** = 14**.

**Model 1**	***r*^2^**	**SEE (BW)**	***P***
	0.432	0.029	0.045
**Independent variables**	**Coefficient**	***t***	**Partial** ***P***
EMG activity of the BF during the end-of-swing phase	0.00061	2.743	0.005
Knee flexors peak torque in concentric mode	0.0499	1.914	0.082
Constant		3.51	0.019
**Model 2**	***r***^2^	**SEE (BW)**	***P***
	0.493	0.028	0.024
**Independent variables**	**Coefficient**	***t***	**Partial** ***P***
EMG activity of the BF during the end-of-swing phase	0.000567	2.67	0.022
Knee flexors peak torque in eccentric mode	0.0384	2.334	0.04
Constant		4.59	<0.001

*SEE: Standard error of estimate*.

A more detailed analysis was conducted distinguishing between the first 10 steps (initial acceleration) and the remaining steps until top speed (end of acceleration). During the initial acceleration phase, similar significant relationships were found between *F*_H_ and the combination of both EMG activity of the BF during the end-of-swing phase and knee flexion peak torque in concentric mode (*P* = 0.038, Table 4). Furthermore, tendencies toward significant relationships were found between *F*_H_ and the combination of Glut EMG activity during the end-of-swing phase and hip extensors peak torque in concentric mode (*P* = 0.041, Table 4).

**Table 4:** **Multiple linear regression analysis for horizontal ground reaction force (in BW) averaged over the first 10 steps (initial acceleration phase, dependent variable in both models), ***n*** = 14**.

**Model 1**	***r*^2^**	**SEE (BW)**	***P***
	0.448	0.036	0.038
**Independent variables**	**Coefficient**	***t***	**Partial** ***P***
EMG activity of the BF during the end-of-swing phase	0.000842	2.759	0.014
Knee flexors peak torque in concentric mode	0.077	2.305	0.042
Constant	0.217	2.904	0.019
**Model 2**	***r***^2^	**SEE (BW)**	***P***
	0.439	0.036	0.041
**Independent variables**	**Coefficient**	***t***	**Partial** ***P***
EMG activity of the Glut during the end-of-swing phase	0.000639	2.638	0.037
Hip extensors peak torque in concentric mode	0.0458	1.908	0.083
Constant	0.244	3.525	0.005

*SEE: Standard error of estimate*.

Finally, no significant relationship or tendency was found between the muscular activity and peak torque variables tested and the values of vertical and resultant forces collected during the sprints.

## Discussion

The aim of this study was to directly investigate the role of hip extensors and knee flexors in horizontal ground reaction force production during sprint running accelerations. The main findings of this study validate our initial hypothesis: the highest level of horizontal ground reaction force production was observed in subjects who had both the highest torque production capability of the hip extensors (especially hamstring muscles in eccentric mode) and the highest hamstring EMG activity during the end-of-swing phase over the entire sprint acceleration. A sub-analysis of the initial acceleration phase (first 10 steps) also showed a significant relationship between horizontal ground reaction force production and glutei concentric torque capability and glutei EMG activity during the end-of-swing phase.

Our initial hypothesis was formulated on the basis of previous studies showing the importance of hamstring force and activation in human running mechanics at top speed, especially during the swing phase (e.g., Kyrölainen et al., 2005; Schache et al., 2011, 2014; Dorn et al., 2012; Sun et al., 2015). In addition, our observation that *F*_H_ differed between elite and less skilled sprinters especially at high running speeds (Morin et al., 2012; Morin, 2013), i.e., when the body is in an overall vertical position, gave support to this hypothesis since the most likely functional possibility to produce high amounts of *F*_H_ is to have a powerful “pawing action” of the (almost fully extended) lower limb prior to ground contact (Mann and Sprague, 1980; Wiemann and Tidow, 1995). Despite some inevitable limitations that will be addressed at the end of this discussion, the main novelty and advantage of the present experimental protocol is that, contrary to previous studies, we could directly investigate the synchronized EMG activity, GRF and 2D motion for all the steps of an entire acceleration phase. Indeed, previous studies gave interesting results, but were based on the analysis of only one or two steps (e.g., Sun et al., 2015), at increasing but constant speeds (e.g., Dorn et al., 2012), and/or used simulation modeling (e.g., Thelen et al., 2005a,b; Fiorentino and Blemker, 2014).

The direct correlations between *F*_H_ averaged over the entire acceleration and the hip extensors peak isokinetic concentric and eccentric torques were not significant. This is certainly due to the angular velocity at which isokinetic tests were performed (120°.s^−1^), which is not realistic compared to the values of hip and knee flexion/extension observed during high-speed running (e.g., >6 times higher values reported by Chapman and Caldwell (1983). In addition, the biarticular hamstring muscles have been shown to contribute to a net transfer of power from proximal to distal joints during explosive leg extensions (Jacobs et al., 1993, 1996), which also might explain the lack of significant correlation between single-joint torque capability and the horizontal force output during a more integrated and functional action such as sprinting. This reinforces the interest of studying muscular force production with realistic joint positions/angles and at the specific velocity of contraction at which it is produced in the actual sport context. Furthermore, the EMG activity of hip extensors (BF and Glut) was not related to *F*_H_, whatever the step cycle phase considered. A tendency (*P* = 0.074) was observed for BF activity averaged over the end-of-swing phase. This result is not surprising either since surface EMG measurements inform about the level of muscular activity, independently from the amount of force a given muscle is able to produce. High EMG activity in a “weak” muscle may not result *in fine* in a high force output. For this reason, we used multiple linear regression models to test the relationship between *F*_H_ as a dependent variable, and two independent variables paired according to our hypothesis (one EMG variable paired with one isokinetic torque variable).

Multiple regression models showed a significant relationship between *F*_H_ and BF EMG activity during the end-of-swing phase, and its torque in concentric (*P* = 0.045) and eccentric (*P* = 0.022) mode (Table 3). This is in line with previous studies using modeling simulation and EMG and/or GRF measurements over a few steps performed at maximal running speed (e.g., Belli et al., 2002; Kyrölainen et al., 2005; Higashihara et al., 2010; Schache et al., 2014), and brings further support to the paramount importance of hamstring muscle force capability (especially in eccentric mode) for acceleration performance. For instance, Figure 3 shows that BF and Glut muscles EMG activity is almost equal to that of VL during the end-of-swing phase. Last and importantly, none of the direct correlation or regression models tested showed significant relationship between any of the muscles considered and the vertical and resultant GRF produced during the acceleration. Similarly, no significant result was found regarding the knee extensor (VL and RF) muscles tested. This study shows that, in the subjects tested (who were all used to perform sprint accelerations), horizontal force production is predominantly determined by hamstring strength and end-of-swing level of activity.

Interestingly, when sub-dividing the entire acceleration phase into the initial acceleration (first 10 steps) and the remaining steps, we observed that Glut EMG activity and peak concentric torque were significantly (regression model *P* = 0.041) related to *F*_H_ averaged over the initial acceleration phase. Some studies highlighted the important role of gluteus muscles, also acting as hip extensors, in acceleration performance (Novacheck, 1998; Belli et al., 2002; Bartlett et al., 2014). Our results confirm this importance, yet only for the initial (first 10 steps) acceleration phase (Table 4). This significant relationship between *F*_H_ and the gluteus concentric strength and activity (end-of-swing phase) only during initial acceleration might be explained by the crouched position on the treadmill only for the very first steps (Figure 4 shows the higher Glut activity in the first half of the stance for the first steps of the acceleration). It is also possible that the subjects tested (who were not sprint experts) were not trained to fully use their glutei muscles during the entire acceleration phase, in comparison with higher-level sprinters. The fact that (i) both hip extensors concentric (Sugiura et al., 2008) and knee flexor eccentric strength (Opar et al., 2015) have been considered risk factors for hamstring strain in different prospective studies and (ii) maximum theoretical *F*_H_ (Mendiguchia et al., 2014) and activity (Daly et al., 2015) are impaired after return to sport from a hamstring injury, support the rationale that both hamstrings and glutei should be considered essential components of any training program for acceleration capabilities. In addition, consequently and logically, these specific hamstrings and glutei strengthening/training programs can also help to prevent posterior thigh injuries (Petersen et al., 2011; Guex and Millet, 2013; Mendiguchia et al., 2015). Since the present results highlight the role of these hip extensor muscles for sprint acceleration performance, weakness in either one may be compensated by the other, as shown in previous modeling studies (Jonkers et al., 2003; Goldberg and Neptune, 2007; Lewis et al., 2009), probably exposing it to a higher risk of injury.

In addition to the aforementioned significant relationships found for the swing and end-of-swing phases, the fact that no significant relationship was found between lower limb muscle force capability and EMG activity during the stance phase (be it only the first half or the entire stance) and *F*_H_ could seem surprising and counterintuitive. Indeed, it could have been expected that muscular force production and activity would be paramount in the horizontally-directed push once the foot is in contact with the ground. This apparent paradox might be explained by the electromechanical delay (EMD) (Cavanagh and Komi, 1979). The delay between the onset of muscle activity (as evidenced here by the EMG data) and the actual onset of force production has been shown to be about 50 ms for hamstring muscles (Novacheck, 1998; Minshull et al., 2007; Hannah et al., 2014). Therefore, since a typical sprint contact phase lasts 150 ms in our study, there is a need for “anticipating” the high hamstring muscular activity during the end-of-swing phase, in order to produce a high backward action force once the foot is in contact with the ground a few tenths of a second later. Alternatively, should the peak of hamstring EMG activity start only once the foot is in contact with the ground, the force induced would likely contribute to *F*_H_ by the end of contact or even later, i.e., too late to be effective for a high *F*_H_ production. Therefore, as Figure 3 shows, and as indicated in previous studies (e.g., Kyrölainen et al., 2005; Chumanov et al., 2011; Higashihara et al., 2014), the high EMG activity of the hamstring muscles during the swing and especially end-of-swing phases of the sprint step cycle (compared to the subsequent stance phase) might induce a more effective backward horizontal push. Similarly, the fact that the eccentric torque capability of the hamstring muscles was more clearly related to *F*_H_ than the concentric one is likely due to the need for a high level of force in order to decelerate the violent knee extension occurring at the end of the swing (Chumanov et al., 2011; Higashihara et al., 2014; Ono et al., 2015), and generate the subsequent powerful backward motion of the lower limb prior to ground contact (and the associated braking GRF) in only a few tenths of a second time.

In this context, we expected that the 2D motion data in the sagittal plane would show a relationship between *F*_H_ and both the high vertical knee position during the swing phase and the backward horizontal velocity of the foot at the end of the swing (Figure 2), i.e., just before ground contact. This has been discussed in some publications (Mann and Sprague, 1980) and it is common practice in athletics training to improve the velocity of the “pawing” or “whipping” backward action of the leg while in the air (end-of-swing phase), with the aim of inducing a higher backward pushing action (i.e., a high *F*_H_) while on the ground. Our data do not support this hypothesis (in the non-expert subjects tested) since no relationship whatsoever was found between 2D kinematic variables of the knee and foot during the swing and end-of-swing phases and subsequent stance phase kinetics, especially *F*_H_. Although further studies are needed to confirm or infirm these results, this puts a very common athletic practice (i.e., training for a violent, quick aerial pawing action of the leg) into question, and could be explained by the fact that the resistance opposing the backward leg action markedly differs between the aerial phase and the ground contact phase. Our results tend to show that a quick backward “whipping” of the leg while in the air does not transfer to a high *F*_H_production during the immediately following stance phase. Although stringing together within a few milliseconds, these two phases are characterized by very different mechanical constraints to the leg motion: high-speed, low resistance (in the air), open kinetic chain vs. lower-speed, high resistance (on the ground), closed kinetic chain. Note that, to our knowledge, no experimental study has brought support to this transfer, and to the link between a fast backward “pawing/whipping” action of the leg and *F*_H_ production. This is, however, the hypothetical basis of many athletic training drills involving fast and intense eccentric actions of the hamstring, e.g., the “B-skip” drill (Puleo and Milroy, 2010).

The multiple-approach experimental protocol we undertook has limitations that must be discussed. First, although it was the only way to study the entire (i.e., all steps) acceleration phase of a sprint and to synchronize GRF, EMG and 2D motion measurements, this study was performed on a sprint instrumented treadmill (Morin et al., 2010). The maximal speed reached and overall sprint performance is lower on such a device, but correlates very well to actual track performance of subjects (Morin and Sève, 2011). In addition, a recent study performed with track-embedded force plates showed that track data of GRF were in line with those obtained on the treadmill for athletes of similar performance level (Rabita et al., 2015). Furthermore, this treadmill protocol did not allow us to test starting-blocks acceleration, but (i) sprint start mechanics have been detailed already (Jacobs and Van Ingen Schenau, 1992; Otsuka et al., 2014) and (ii) our conclusions will likely apply to sprinting in addition to other sports whereby sprint accelerations start in a standing or slightly crouched position (e.g., soccer, rugby), which is the case on the sprint treadmill. Finally, the fact that the treadmill was motorized might have induced a slight external assistance to the stance backward action of the leg and altered our EMG results during this specific phase. However, we think this is unlikely because (i) the *F*_H_ values and power output we report here are consistent with those obtained on the track by other authors (e.g., Rabita et al., 2015), and (ii) we showed in a treadmill-track comparison that within the same subjects, the *F*_H_ values produced on the treadmill were even higher than on the track (Morin and Sève, 2011), which tends to cast doubt on this limit. In addition, only motorized treadmills can allow mechanical sprinting conditions close to actual practice, as discussed by McKenna and Riches (2007).

Second, we measured the EMG activity of the BF muscles, which is only a part of the hamstring group. Since recent studies showed the different activation sequences among the muscles of the hamstring group during sprint running (Higashihara et al., 2010, 2015; Nagano et al., 2014), one should be careful when extending the results of the BF to the more general term “hamstring.” In addition, only the right leg was studied for technical reasons, but we have no strong evidence or argument not to expect that both legs behaved similarly in this context.

Finally, our methods included surface EMG and isokinetic testing as a way to experimentally inform on the level of muscular activity during the sprint, and on the global level of (eccentric and concentric) torque capability of the muscles groups tested, respectively. This is of course less accurate than direct measurements of activation and force at the muscle level, and does not reproduce sprint-specific conditions (e.g., knee flexion tested in marked hip extension while seated), but those measurements over the entire course of maximal sprint accelerations are currently not possible, hence the numerous modeling simulation studies published on this topic (e.g., Ono et al., 2015).

## Conclusion

This study showed that, in individuals familiar with sprinting, a greater amount of horizontal GRF (as averaged over an entire sprint acceleration) was found in subjects who were both able to highly activate their hamstring muscles just before ground contact and had the greatest capacity to produce eccentric hamstring torque. In other words, hamstring EMG activity during the swing and end-of-the-swing phases and eccentric knee flexor peak torque are related to the amount of horizontal GRF produced during sprinting, likely because of the backward “pawing” action of the leg just before contact. These findings suggest that, in addition to their paramount importance in sprint-related sports injuries, the hip extensors, and especially the hamstrings, play a significant role in sprint acceleration performance via horizontal GRF production.

## Perspectives

One of the most important research perspectives following this cross-sectional study is to consider the effects of training programs aimed at reinforcing hip extensor strength, and hamstring strength in particular (including the eccentric action mode) on sprint acceleration performance. Although such training programs are considered of interest for hamstring injury prevention, this study clearly makes the hypothesis of their additional interest for sprint performance worth considering. Indeed, working on hamstring muscle strength (and particularly in eccentric mode) has been proposed as a potentially efficient prevention approach (e.g., Petersen et al., 2011; Schmitt et al., 2012; Askling et al., 2013, 2014; Guex and Millet, 2013; Bizzini and Dvorak, 2015). This study brings novel insights into the fact that hip extensors, especially hamstring muscles, must be carefully considered by training practitioners for their role in sprint acceleration performance, in addition to their unique place regarding injury risk and prevention. One practical application of the present results is that increasing eccentric knee flexion strength along with concentric hip extension strength could also be an efficient approach to improving sprint acceleration performance. Finally, since repeated sprint and the associated hamstring fatigue is a key feature in team sports such as rugby or soccer (Small et al., 2009; Greco et al., 2013; Marshall et al., 2014), it could be interesting to investigate such fatigued conditions.

## Author contributions

Conceived and designed the experiments: JM, PE, PS, MB, JM. Performed experiments: JM, PG, PE, PR, PS. Analyzed data: JM, PG, PE, PA. Interpreted results of research: JM, PG, PA, PS, MB, JM. Drafted manuscript and prepared tables/figures: JM, PA, PE. Edited, critically revised paper and approved final version of manuscript: JM, PG, PE, PA, PR, PS, MB, JM.

### Conflict of interest statement

The authors declare that the research was conducted in the absence of any commercial or financial relationships that could be construed as a potential conflict of interest.
